# High human papillomavirus (HPV)-35 prevalence among South African women with cervical intraepithelial neoplasia warrants attention

**DOI:** 10.1371/journal.pone.0264498

**Published:** 2022-03-09

**Authors:** Zizipho Z. A. Mbulawa, Keletso Phohlo, Mirta Garcia-Jardon, Anna-Lise Williamson, Charles B. Businge

**Affiliations:** 1 National Health Laboratory Service, Nelson Mandela Academic Hospital, Mthatha, South Africa; 2 Faculty of Health Sciences, Department of Laboratory Medicine and Pathology, Walter Sisulu University, Mthatha, South Africa; 3 UCT-MRC Gynaecological Cancer Research Centre, University of Cape Town, Cape Town, South Africa; 4 Division of Medical Virology, Department of Pathology, University of Cape Town, Cape Town, South Africa; 5 Institute of Infectious Disease and Molecular Medicine, University of Cape Town, Cape Town, South Africa; 6 Department of Obstetrics and Gynaecology, Nelson Mandela Academic Hospital, Mthatha, South Africa; 7 Faculty of Health Sciences, Department of Obstetrics and Gynaecology, Walter Sisulu University, Mthatha, South Africa; Istituto Nazionale Tumori IRCCS Fondazione Pascale, ITALY

## Abstract

Human papillomavirus (HPV) prevalence and genotype distribution data is important for HPV vaccine monitoring. This study investigated the prevalence and distribution of HPV genotypes in cervical lesions of unvaccinated women referred to Nelson Mandela Academic Hospital Gynaecology Department due to different abnormal cervical conditions. A total of 459 women referred to the Nelson Mandela Academic Hospital Gynaecology department were recruited. When the cervical biopsy was collected for histopathology, an adjacent biopsy was provided for HPV detection. Roche Linear Array HPV genotyping assay that detects 37 HPV genotypes was used to detect HPV infection in cervical biopsies. HPV infection was detected in 84.2% (383/455) of participants. The six most dominant HPV types were HPV-16 (34.7%), followed by HPV-35 (17.4%), HPV-58 (12.1%), HPV-45 (11.6%), HPV-18 (11.4%) and HPV-52 (9.7%). HPV-35 was the third most dominant type among women with cervical intraepithelial lesion (CIN)-2 (12.6%; single infection: 5.7% and multiple infection: 6.9%), the second most dominant type among women with CIN3 (22.2%; single infection: 8.0% and multiple infection: 14.2%); and the fourth most dominant type among women with cervical cancer (12.5%; single infection: 7.1% and multiple infection: 5.4%). A proportion of 41.1% (187/455) was positive for HPV types targeted by the Cervarix®, 42.4% (193/455) by Gardasil®4, and 66.6% (303/455) by Gardasil®9. There was a statistically significant increase when the prevalence of women infected with HPV-35 only or with other HPV types other than Gardasil®9 types was included to those infected with Gardasil®9 HPV types (66.6%, 303/455 increase to 76.0%, 346/455, p = 0.002). High HPV-35 prevalence in this population, especially among women with CIN3 warrants attention since it is not included in current commercially available HPV vaccines.

## Introduction

Cervical cancer is the second most common cancer in South African women [1]. The National Cancer Registry (NCR) of South Africa reported an age-standardized rate (ASR) of 22.56 per 100,000 for all South African women in 2014 and an ASR of 27.01 per 100,000 for black African women [2]. According to Somdyala et al. (2020), in the rural Eastern Cape Province of South Africa, the annual cervical cancer ASR per 100,000 increased from 22.0 in 1998–2002 to 29.2 in 2008–2012 [3]. Of the African countries, South Africa has the largest population affected by human immunodeficiency virus (HIV) infection, with 7.8 million people living with HIV and 230 000 new infections reported in 2020 [4]. Both cervical cancer and HIV burden are high in African countries [5–7].

Human papillomavirus (HPV) is the most common sexually transmitted virus [8–11], with its peak prevalence observed in adolescents and young women soon after sexual debut and decreasing with increasing age in women [12,13]. Persistent infection with HR-HPV genotypes is associated with the development of cervical lesions and cervical cancer [14]. Compared to HIV-negative individuals, the HIV-infected individuals are more likely to be infected by HPV, co-infected with multiple HPV types, persistent infection, reactivation, and develop HPV-associated cancers on different anatomical sites [7,15–18]. Among HPV types known to be dominant in cervical cancer cases worldwide, HPV-16 is the most common type worldwide, followed by HPV-18 [19–21]. It is important to note that among women of African ancestry origin, HPV-35 is detected in approximately 10% of cervical cancer cases, while it is detected in approximately 2% of worldwide cases [1,21,22]. Studies in Sub-Saharan African populations have reported an HPV-35 prevalence of up to 40% among women with cervical intraepithelial neoplasia (CIN) or cervical cancer [21,31,35–38].

Currently, there are three HPV vaccines approved by the U.S Food and Drug Administration. They are Cervarix® (GlaxoSmithKline), Gardasil®4 (Merck Inc), and Gardasil®9 (Merck Inc). They target a different combination of HPV types; Cervarix® targets HPV-16/18, the most carcinogenic HPV types associated with approximately 70% cervical cancer cases; Gardasil®4 targets HPV-16/18 as well as two low-risk (LR) types, HPV-6/11, associated with genital warts and recurrent respiratory papillomatosis; and Gardasil®9 (Merck Inc) targets same types as Gardasil®4 and the other five high-risk (HR) HPV types HPV-31/33/45/52/58 [6,23–25]. Sub-Saharan African HIV-positive women were reported to have decreased HPV types targeted by Gardasil®9 HPV vaccine (HPV-6/11/16/18/31/33/45/52/58) when compared with Sweden-born HIV-negative and HIV-positive women. These observations were influenced by decreased HPV-16 and increased HPV-35 prevalence in sub-Saharan African women [26]. Unfortunately, the currently commercially available HPV vaccines are not targeting HPV-35 [6].

Many countries have implemented HPV vaccination programs since the first licensure of Gardasil®4 in 2006 [24,25]. In South Africa, the school-based national HPV vaccination program was introduced in 2014, targeting girls aged nine years or older (mostly in grade-4), and the Cervarix® HPV vaccine two-dose schedule is used in this program. The vaccine schedules are 6-months apart within the academic calendar year [23,27,28]. Different strategies are implemented to prevent pre-invasive lesions and cervical cancer, mainly through HPV vaccination and cervical cancer screening [29].

As part of the HPV vaccination strategy in South Africa, it is essential to have information on HPV prevalence and HPV types distribution among the unvaccinated population to inform vaccination campaigns and monitor the impact on HPV types after vaccination [30]. The data needs to come from population-based surveillance and women with cervical disease in unvaccinated women. Information on the prevalence of HPV and the distribution of HPV types in women residing in the Eastern Cape Province of South Africa is limited [31] and based on cervical specimens collected by cervical brush. The current report presents HPV genotyping data in cervical biopsies and this is significant because HPV types detected in cervical biopsies are more likely to be integrated into tissue and associated with the observed lesion. In contrast, in cytobrush specimens, all the HPV genotypes that are on the cervix will be detected including transient types [32,33]. Therefore, this study aimed to investigate the prevalence and distribution of HPV genotypes in cervical biopsy of HPV unvaccinated women referred to Gynaecology Department Nelson Mandela Academy Hospital in Mthatha, Eastern Cape.

## Materials and methods

### Ethical statement

Participation in the study was voluntary, with written informed consent. This study was approved by the Human Research Ethics Committees of the University of Cape Town (HREC: 079/2014) and Walter Sisulu University (reference: 090/2016). Permission to conduct research in the Eastern Cape was granted by the Eastern Cape Provincial Health Research Committee (EC_2016RP29_562). Participation in the study was voluntary, with written informed consent.

### Study setting, population, and recruitment

This project is a hospital-based project among women with high-grade cervical lesions and cervical cancer referred to Nelson Mandela Academy Hospital in Mthatha, Eastern Cape. Nelson Mandela Academic Hospital serves most of the population residing in the former Transkei region of the Eastern Cape Province (OR Tambo, Chis Hani, Alfred Nzo, Amathole, and Joe Gqabi municipality). Between February 2018 and March 2020, women aged ≥18 years with atypical squamous cells cannot exclude high-grade lesions (ASC-H), low-grade squamous intraepithelial lesions (LSIL), high-grade squamous intraepithelial lesions (HSIL), and cervical cancer referred to Nelson Mandela Academic Hospital Gynaecology department were recruited. When the cervical biopsy was collected for histopathology, an adjacent piece was provided for HPV detection. Histopathology was conducted by National Health Laboratory Service, Histopathology Laboratory at the Nelson Mandela Academic Hospital. Cervical biopsy for HPV detection was stored in Digene transport medium at -20°C and transported to the University of Cape Town, HPV Laboratory.

### Nucleic acid extraction and HPV detection

A cervical biopsy specimen was lysed using MagNA Pure 96 tissue lysis buffer. Nucleic acid was extracted using an automated procedure of MagNA Pure Compact (Roche Molecular Systems, Inc., Branchburg, NJ, USA) and MagNA Pure Compact Nucleic Acid Isolation Kit (Roche Molecular Systems, Inc., Branchburg, NJ, USA). Roche Linear Array HPV Genotyping Test (Roche Molecular Systems, Inc., Branchburg, NJ, USA) was used to detect HPV genotypes in extracted nucleic acid from cervical biopsy specimens and manufacturer instructions were followed. The Linear Array HPV Genotyping Test amplifies the target HPV DNA for 37 anogenital HPV genotypes, namely, HPV-6, -11, -16, -18, -26, -31, -33, -35, -39, -40, -42, -45, -51, -52, -53, -54, -55, -56, -58, -59, -61, -62, -64, -66, -67, -68, -69, -70, -71, -72, -73, -81, -82, -83, -84, -IS39 and -CP6108 (HPV-89). The Linear Array HPV Genotyping Test also amplifies the β-globin gene to monitor sample adequacy, extraction, amplification, and hybridization.

### Data analysis

All variables were captured and coded in Microsoft Excel 2013. Participants were counted more than once when determining the prevalence of LR-HPV, HR-HPV, and probable HR-HPV if they have types that belong to more than one category. Single infection was defined as infection with one HPV type. Multiple HPV infections were defined as the detection of two or more HPV types in the same sample. Statistical analysis was performed using chi-squared for trends and Fisher’s exact for comparison of the HPV prevalence (GraphPad Prism Software v6.01). The level of significance was set at 5% (p-value ≤ 0.05) for statistical significance.

## Results

### Demographic characteristics of study participants

A total of 459 women were recruited. Four samples were negative for the β-globin gene and were therefore excluded from the analysis. Study participants were between the ages of 18 and 90 years, with a median of 42 years. The majority of study participants were HIV-positive (65.7%, 299/455), and 88.3% were on ARVs. Only 19.8 (90/455) had training after high school education. A higher proportion of study participants had three to four lifetime sexual partners (Table 1).

**Table 1:** Demographic and behavioural characteristics of study participants.

Characteristics	%	n/N
**Age in years, median (range)**		42 (18–90)
**Age group**		
18–30 years	5.9	27
31–50 years	32.3	147
51–90 years	61.8	281
**HIV status**		
positive	65.7	299/455
negative	27.5	125/455
missing	6.8	31/455
**If HIV-positive, on ARVs**		
Yes	88.3	264/299
No	11.7	35/299
**Education**		
Never	4.6	21/455
Primary school (Grade 1–7)	28.6	130/455
High school (Grade 8–12)	47.0	214/455
University	19.8	90/455
**Age at first sex**		
≤16 years	24.4	111/455
17–18 years	31.9	145/455
19–20 years	31.2	142/455
21–33 years	12.5	57/455
**Lifetime sexual partners**		
1–2	28.8	131/455
3–4	55.2	251/455
5–10	16.0	73/455
**Cervical cytology**		
ASC-H	8.8	40/455
LSIL	5.5	25/455
HSIL	81.5	371/455
Cervical cancer	4.2	19/455
**Cervical histology**		
CIN1	2.2	10/455
CIN2	19.1	87/455
CIN3	38.7	176/455
Cervical cancer	12.3	56/455
No dysplasia	9.2	42/455
No results	17.8	81/455
Poor quality specimens	0.7	3/455

### HPV prevalence and genotype distribution

HPV infection was detected in 84.2% (383/455) of women. The majority of participants were infected with HR-HPV type(s) (80.2%, 365/455). While only 8.1% (37/455) were infected with probable HR-HPV type(s), and 15.4% (70/455) with LR-HPV type(s). Infection with single HPV type (46.6%, 212/455) was common than multiple HPV infections (37.6%, 171/455, p = 0.007) in this population (Table 2). The overall HPV prevalence remained high in both HIV-positive women (88.3%, 264/299) and HIV-negative (80.0%, 100/125, p = 0.063). When stratified according to carcinogenicity level, the prevalence of multiple infection, HR-HPV, and LR-HPV was found to be significantly high among HIV-positives compared to HIV-negative individuals (p = 0.022, p = 0.038, and p = 0.025 respectively, Table 2).

**Table 2:** Prevalence of HPV infection according to HIV status among women referred to Nelson Mandela Academic Hospital Gynaecology department, Eastern Cape Province.

Variables	All participants, N455	HIV-negative, N = 125	HIV-positive, N = 299	No HIV status, N = 31	p-value*
Any types	84.2%, 383/455	80.0%, 100/125	88.3%, 264/299	61.3%, 19/31	0.063
Single infection	46.6%, 212/455	49.6%, 62/125	45.8%, 137/299	41.9%, 13/31	0.522
Multiple infection	37.6%, 171/455	30.4%, 38/125	42.5%, 127/299	19.4%, 6/31	**0.022**
HR-HPV types	80.2%, 365/455	76.0%, 95/125	84.6%, 253/299	54.8%, 17/31	**0.038**
Probable HR-HPV types	8.1%, 37/455	40.0%, 5/125	10.4%, 31/299	3.2%, 1/31	**0.035**
LR-HPV	15.4%, 70/455	8.8%, 11/125	17.7%, 53/299	19.4%, 6/31	**0.025**

*compares HIV–negative and positive prevalence. **HR–HPV**: High–risk human papillomavirus; **LR–HPV**: Low–risk human papillomavirus; **HR–HPV types**: HPV–16, –18, –31, –33, –35, –39, –45, –51, –52, –56, –58 and –59. **Probable HR–HPV types**: HPV–26, –53, –66, –67, –68, –70, –73 and –82. **LR–HPV**: HPV–6, –11, –40, 42, –54, –55, –61, –62, –64, –69, –71, –72, –81, –83, –84, –89 (CP6108) and–IS39.

**Fig 1:**
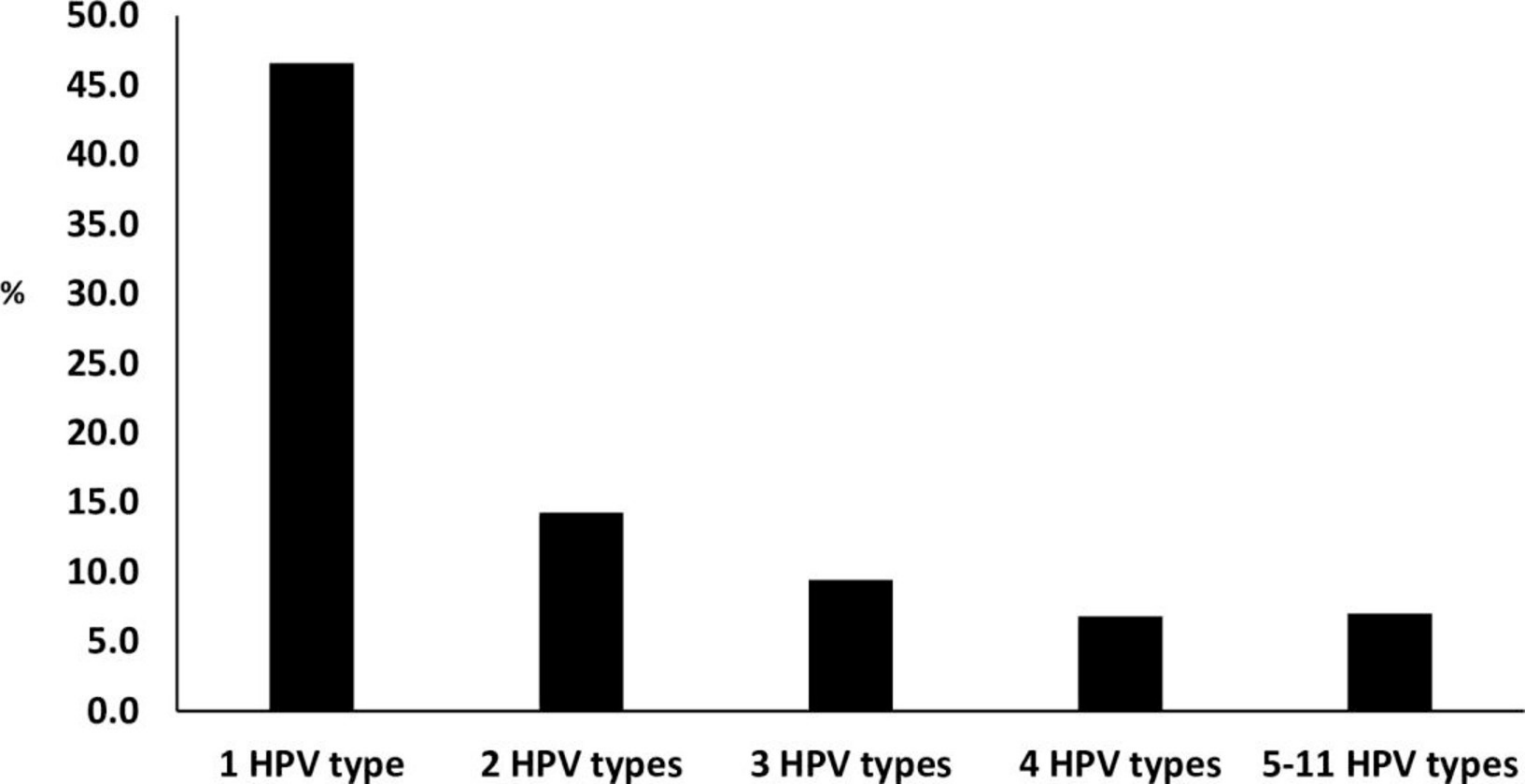
Human papillomavirus infection among women referred to Nelson Mandela Academic Hospital Gynaecology department, Eastern Cape Province.

**Fig 2:**
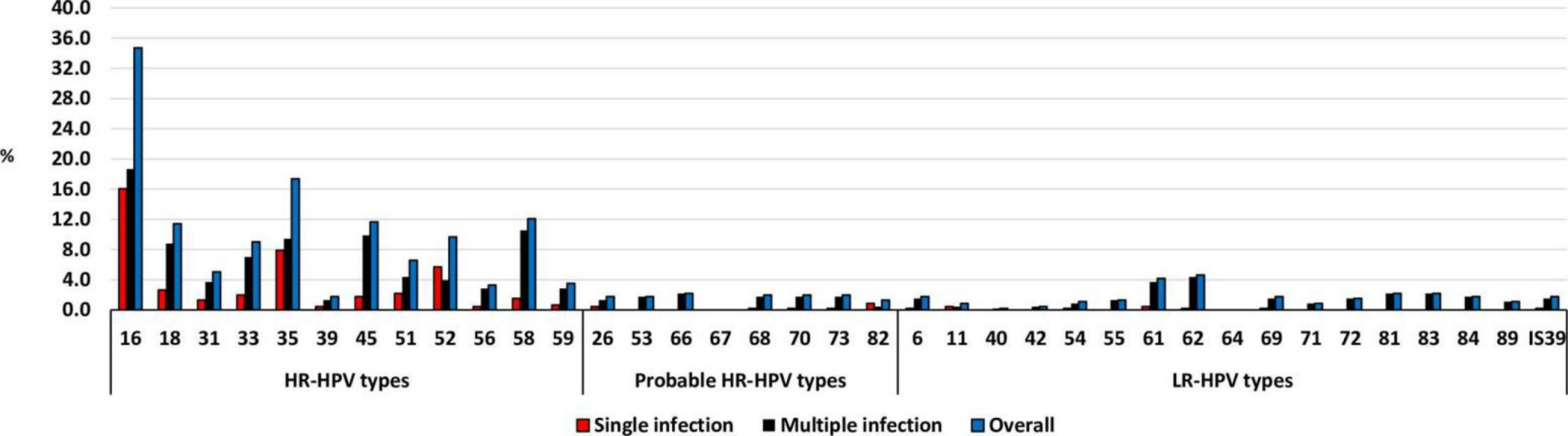
Human papillomavirus genotype distribution among women referred to Nelson Mandela Academic Hospital Gynaecology department, Eastern Cape Province. Single HPV infection is defined as infection with one HPV type, while multiple HPV infections as the detection of two or more HPV types in the same sample.

A proportion of 46.6% (212/455) were infected with one HPV type, 14.3% (65/455) were infected with two different HPV types, 9.5% (43/455) were infected with three different HPV types, 6.8% (31/455) were infected with four different HPV types, and 7.0% (32/455) were infected with five to eleven different HPV types (Fig 1). The distribution of HPV types detected among participants is presented in Fig 2. The six most dominant HPV types were HPV-16 (34.7%), followed by HPV-35 (17.4%), HPV-58 (12.1%), HPV-45 (11.6%), HPV-18 (11.4%) and HPV-52 (9.7%, Fig 2). When focusing on dominant HPV types that were detected as a single infection, HPV-16 remains the most dominant type, followed by HPV-35 and HPV-52 (Fig 2).

### HPV prevalence according to cervical histology data

A proportion of 98.2% (55/56) women with cervical cancer were HPV infected, and infection with a single HPV type was more common than multiple infections (58.9% 33/56; 39.3% 22/56, p = 0.058). Of women with CIN-3, 89.7% (157/175) were HPV infected, 85.1% (74/87) CIN-2 women, and 50.0% (5/10) CIN-1 (Table 3). HR-HPV types were common, and their prevalence was increasing with disease severity, CIN2 (79.3%, 69/87), CIN3 (86.9%, 153/176), and cervical cancer (96.4%, 54/56, p = 0.003). The HPV prevalence was found to be higher among the HIV-positive women with CIN2 (92.2%, 59/64) compared to HIV-negatives (65.2%, 15/23, p = 0.004, Table 3).

**Table 3:** Human papillomavirus prevalence according to cervical histology data among women referred to Nelson Mandela Academic Hospital Gynaecology department, Eastern Cape Province.

	All		HIV-positive		HIV-negative		p-value
Variable	%	n/N	%	n/N	%	n/N	
**CIN2, N = 87**							
Any HPV infection	85.1	74/87	92.2	59/64	65.2	15/23	**0.004**
Single HPV infection	47.1	41/87	50.0	32/64	39.1	9/23	0.467
Multiple HPV infection	37.9	33/87	42.2	27/64	26.1	6/23	0.215
HR-HPV types	79.3	69/87	87.5	56/64	56.5	13/23	**0.005**
*1HR-HPV type*	*53*.*6*	*37/69*	*51*.*8*	*29/56*	*61*.*5*	*8/13*	*0*.*556*
*≥2HR-HPV types*	*17*.*4*	*12/69*	*21*.*4*	*12/56*	*0*.*0*	*0/13*	*0*.*104*
*1HR-HPV & other types*	*11*.*6*	*8/69*	*10*.*7*	*6/56*	*15*.*4*	*2/13*	*0*.*639*
*≥2HR-HPV & other types*	*17*.*4*	*12/69*	*16*.*1*	*9/56*	*23*.*1*	*3/13*	*0*.*685*
**CIN3, N = 176**							
Any HPV infection	89.8	158/176	90.7	116/129	87.2	41/47	0.592
Single HPV infection	48.3	85/176	45.7	58/129	55.3	26/47	0.401
Multiple HPV infection	41.5	73/176	45.0	58/129	31.9	15/47	0.237
HR-HPV types	86.9	153/176	86.8	111/129	87.2	41/47	1.000
*1HR-HPV type*	*53*.*6*	*82/153*	*50*.*0*	*55/111*	*63*.*4*	*26/41*	*0*.*146*
*≥2HR-HPV types*	*22*.*9*	*35/153*	*22*.*3*	*25/111*	*24*.*4*	*10/41*	*0*.*830*
*1HR-HPV & other types*	*9*.*8*	*15/153*	*13*.*4*	*15/111*	*0*.*0*	*0/41*	***0*.*012***
*≥2HR-HPV & other types*	*13*.*7*	*21/153*	*14*.*3*	*16/111*	*12*.*2*	*5/41*	*1*.*000*
**Cervical cancer, N = 56**							
Any HPV infection	98.2	55/56	100.0	33/33	95.5	21/22	0.400
Single HPV infection	58.9	33/56	54.5	18/33	63.6	14/22	0.583
Multiple HPV infection	39.3	22/56	45.5	15/33	31.8	7/22	0.403
HR-HPV types	96.4	54/56	97.0	32/33	90.9	21/22	1.000
*1HR-HPV type*	*59*.*3*	*32/54*	*53*.*1*	*17/32*	*66*.*7*	*14/21*	*0*.*300*
*≥2HR-HPV types*	*33*.*3*	*18/54*	*34*.*4*	*11/32*	*33*.*3*	*7/21*	*1*.*000*
*1HR-HPV & other types*	*1*.*9*	*1/54*	*3*.*1*	*1/32*	*0*.*0*	*0/21*	*1*.*000*
*≥2HR-HPV & other types*	*5*.*6*	*3/54*	*9*.*4*	*3/32*	*0*.*0*	*0/21*	0.*269*

*compares HIV–negative and positive prevalence. Single HPV infection is defined as infection with one HPV type, while multiple HPV infections as the detection of two or more HPV types in the same sample. **HR–HPV**: High–risk human papillomavirus; **LR–HPV**: Low–risk human papillomavirus; **HR–HPV types**: HPV–16, –18, –31, –33, –35, –39, –45, –51, –52, –56, –58 and –59. **Probable HR–HPV types**: HPV–26, –53, –66, –67, –68, –70, –73 and –82. **LR–HPV**: HPV–6, –11, –40, 42, –54, –55, –61, –62, –64, –69, –71, –72, –81, –83, –84, –89 (CP6108) and–IS39.

HIV–positive and HIV–negative participants do not always add up to all women because there were participants with unknown HIV status.

**Fig 3:**
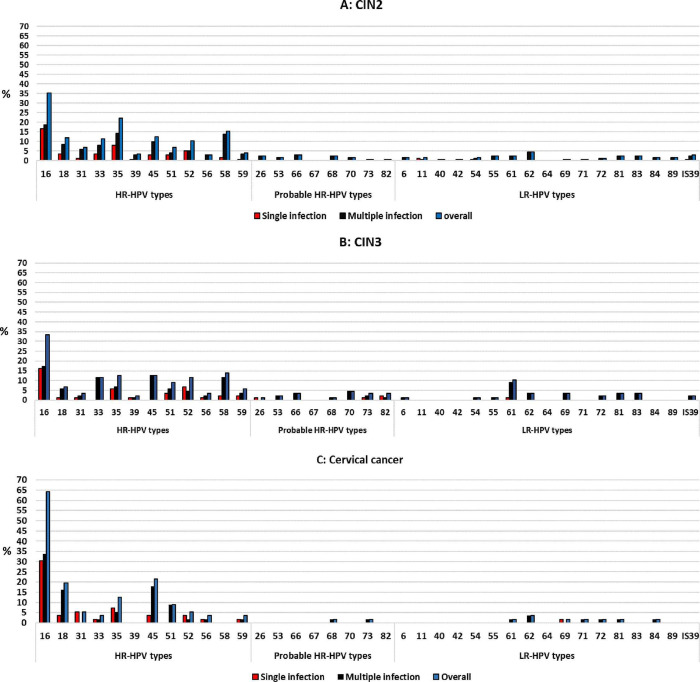
High–risk human papillomavirus genotype distribution among women with CIN2 (A), CIN3 (B) or cervical cancer (C) referred to Nelson Mandela Academic Hospital Gynaecology department, Eastern Cape Province according to cervical histology data.

Among women with cervical cancer, HPV-16 (64.3%) was the most dominant type, followed by HPV-45 (21.4%), HPV-18 (19.6%), and HPV-35 (12.5%). Among women with CIN3, HPV-16 (35.2%) was the most dominant type, followed by HPV-35 (22.2%), HPV-58 (15.3%), and HPV-45 (12.5%). Among women with CIN2, HPV-16 (33.3%) remain the most dominant type, followed by HPV-58 (13.8%), HPV-35 (12.6%), and HPV-45 (12.6%, Fig 3).

Infection with a single HPV type (47.1%, 41/87) was more common than infection with multiple HPV types (37.9%, 33/87, p = 0.283) among women with CIN2 (Table 3). When focusing on the frequency of each HPV type detected in multiple infection, it was found to be between 1.1% and 11.5% among the HR-HPV types; and 0.0% - 9.2% for the LR/probable-HPV types. While among single infection, it was found to range between 0.0% and 16.1% among the HR-HPV types; and 0.0% and 2.3% for the LR/probable-HPV types (Fig 3A).

Infection with a single HPV type (48.3%, 85/176) was more common than multiple HPV infections (41.5%, 73/176, p = 0.238) among women with CIN3 (Table 3). When focusing on the frequency of each HPV type detected in multiple infection, it was found to be between 2.8% and 18.8% among the HR-HPV types; and 0.0% - 4.5% for the LR/probable-HPV types. While among single infection, it was found to range between 0.0% and 16.5% among the HR-HPV types; and 0.0% and 0.6% for the LR/probable-HPV types (Fig 3B).

Infection with a single HPV type (58.9%, 33/56) was more common than infection with multiple HPV types (39.3%, 22/56, p = 0.058) among women with cervical cancer (Table 3). When focusing on the frequency of each HPV type detected in multiple infections, it was found to be between 0.0% and 33.9% among the HR-HPV types; and 0.0% and 3.6% for the LR/probable-HPV types. While among single infection, it was found to range between 0.0% and 30.4% among the HR-HPV types; and 0.0% and 1.8% for the LR/probable-HPV types (Fig 3C).

### HPV type distribution and prevalence of HPV types targeted by HPV vaccines

**Fig 4:**
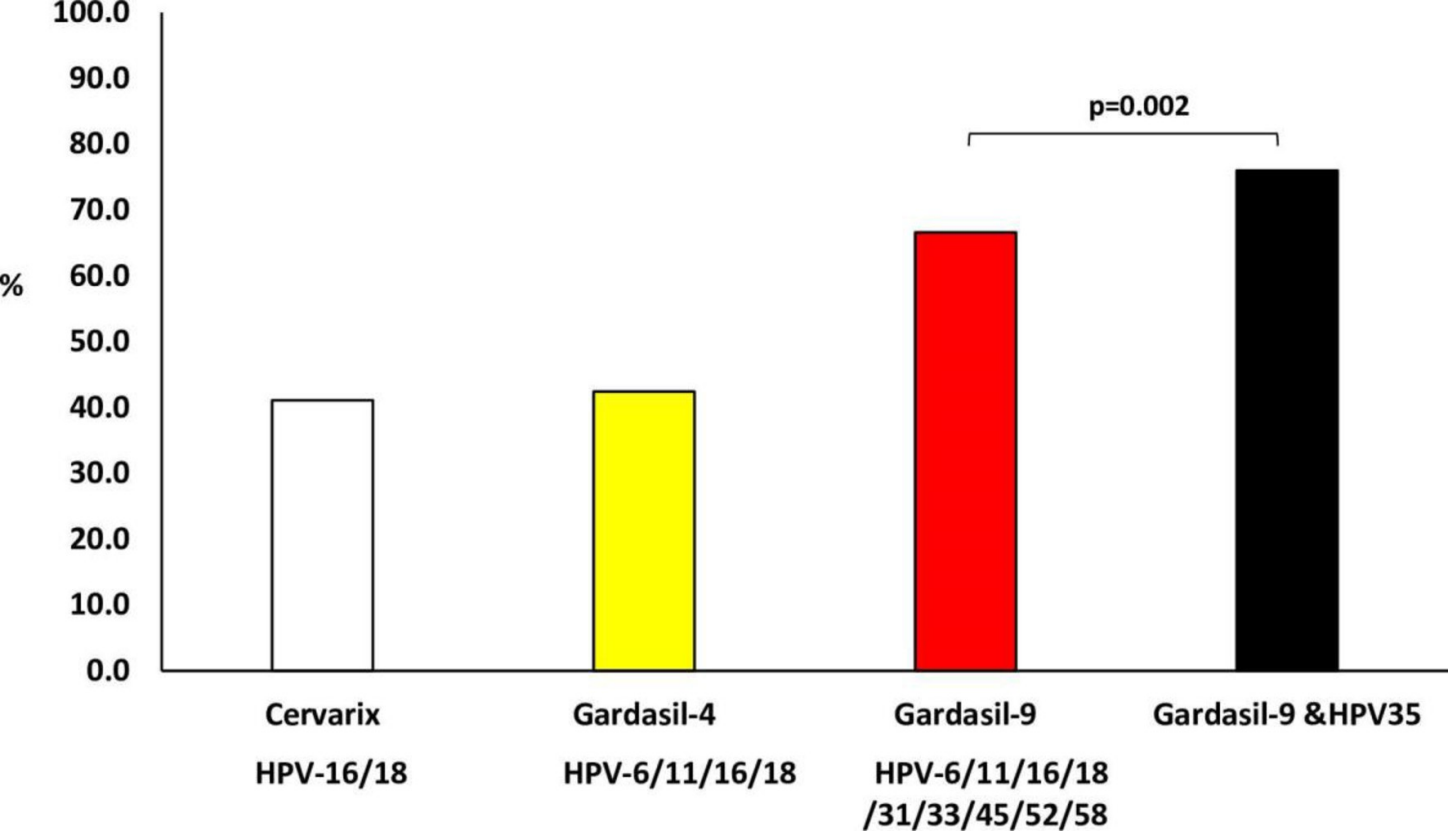
Prevalence of Human papillomavirus (HPV) types targeted by current commercial HPV vaccines among women referred to Nelson Mandela Academic Hospital Gynaecology Department, Eastern Cape. (Cervarix vaccine targets HPV–16/18; Gardasil–4 vaccine targets HPV–6/11/16/18 and Gardasil–9 vaccine targets HPV–6/11/16/18/31/33/45/52/58).

HPV type(s) targeted by the Cervarix® HPV vaccine (HPV-16 and/or 18), currently used in the South African school-based HPV vaccination program, were detected in 41.1% (187/455), those targeted by Gardasil®4 (HPV-6, -11, -16 and/or -18) were detected in 42.4% (193/455), and those targeted by Gardasil®9 (HPV-6, -11, -16, -18, -31, -33, -45, -52 and/or -58) were detected in 66.6% (303/455, Fig 4).

**Fig 5:**
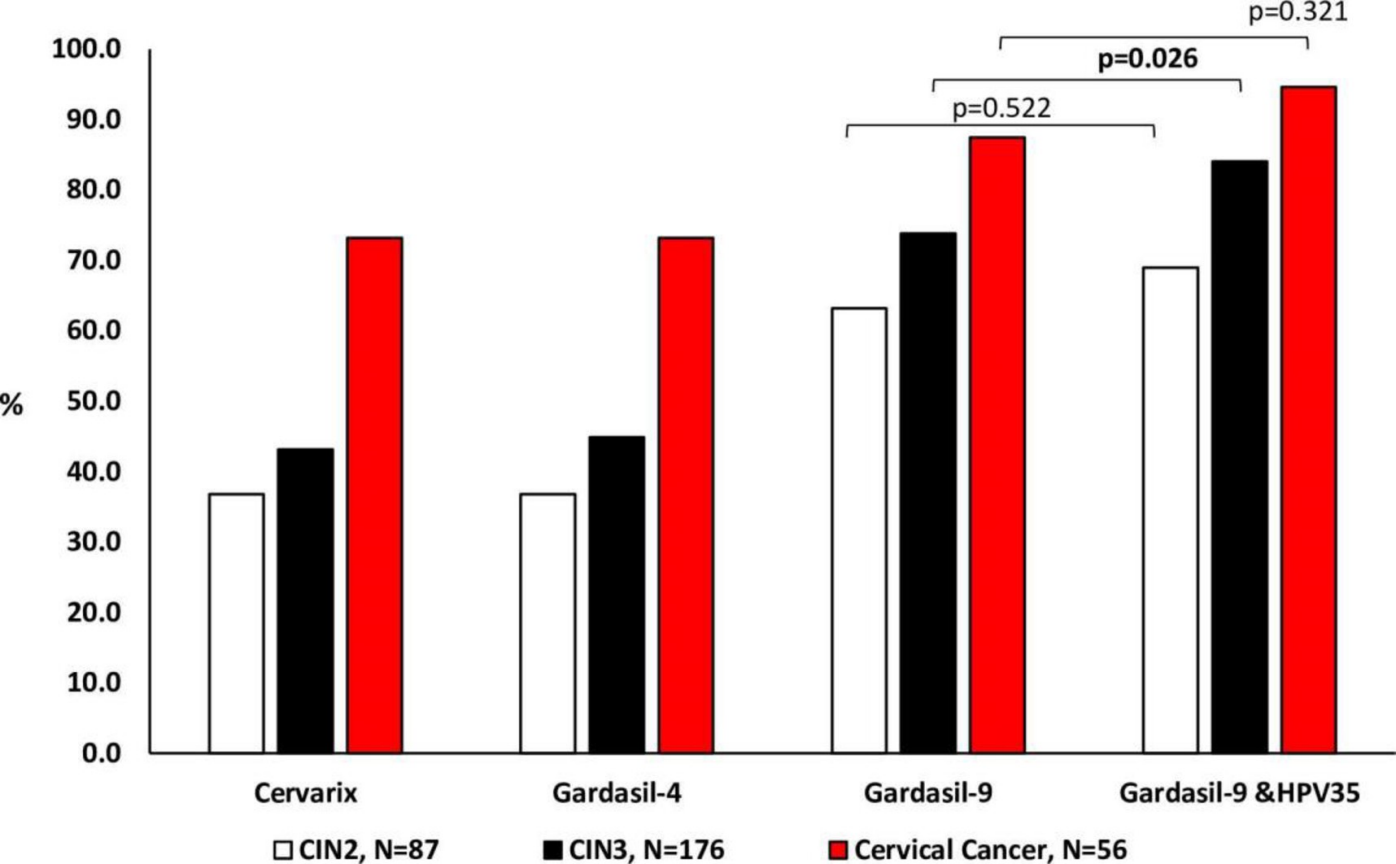
Prevalence of Human papillomavirus (HPV) types targeted by current commercial HPV vaccines among women according to cervical disease status (Cervarix vaccine targets HPV–16/18; Gardasil–4 vaccine targets HPV–6/11/16/18 and Gardasil–9 vaccine targets HPV–6/11/16/18/31/33/45/52/58).

When stratified according to cervical disease status, types targeted by the Cervarix® HPV vaccine were detected in 36.8% (32/87) women with CIN2, 43.2% (76.176) women with CIN3, and 73.2% (41/56) women with cervical cancer. Types targeted by the Gardasil®4 HPV vaccine were detected in 36.8% (32/87) women with CIN2, 44.9% (79/176) women with CIN3, and 73.2% (41/56) women with cervical cancer. Types targeted by Gardasil®9 HPV vaccine were detected in 63.2% (55/87) women with CIN2, 73.9% (130/176) women with CIN3, and 87.5% (49/56) women with cervical cancer (Fig 5).

### HPV-35 prevalence and distribution according to cervical histology diagnosis

HPV-35 was the second most common HPV type detected (17.4%), single infection was observed in 7.9% and multiple infection in 9.5% (Fig 2). Among women with CIN2, HPV-35 was the third most dominant type (12.6%), single infection was observed in 5.7% and multiple infection in 6.9%. While among women with CIN3, HPV-35 was the second most dominant type (22.2%), single infection was in 8.0% and multiple infection in 14.2%. HPV-35 was the fourth most common type among women with cervical cancer (12.5%), single infection was observed in 7.1% and multiple infection in 5.4% (Fig 3).

There was a statistically significant increase when the prevalence of women infected with HPV-35 only or with other HPV types other than those targeted by Gardasil®9 was included among those infected with Gardasil®9 HPV types (66.6%, 303/455 increase to 76.0%, 346/455, p = 0.002) in the overall population. When participants were grouped according to histology, this increase was also observed among women with CIN3 (73.9%, 130/176 increase to 84.1%, 148/176, p = 0.026). However, there was no significant increase among CIN2 (63.2%, 55/87; 69.0%, 60/87, p = 0.522) and cervical cancer (87.5%, 49/56; 94.6%, 53/56, p = 0.321, Fig 5).

## Discussion

This study investigated the cervical HPV prevalence and distribution among women referred to the hospital with cervical disease. As expected, the overall HPV (84.2%) was high in the study population. However, the overall HPV prevalence among women with CIN2 (85% compared to 93%) and CIN3 (89% compared to 97%) was lower than the study recently reported by Taku et al. in the same district as the current report [31]. The observed low overall HPV prevalence and that of individual types in the current study could be affected by specimen type and collection. The cervical biopsy was used in the current study for HPV detection, while the cervical swab/brush collected from the whole cervix was used in the study by Taku et al. [31]. When the cervical biopsy was collected for histopathology analysis, an adjacent piece was provided for HPV detection; it is possible that the biopsy for HPV detection was not part of the lesion. Two cervical cancer cases were negative for HR-HPV infection and could be an indication of false-negative result due to the integration of HPV which resulted in disruption or loss of the primer targeted sequence. It is also possible that the HPV DNA was absent in the collected cervical biopsy [32].

The majority of the observed single infections appeared as HR-HPV types. Few LR-HPV types were detected as a single infection and their prevalence was low (0.4%). It is possible that the probable HR or HR-HPV type(s) responsible for the observed cervical lesion was mis-detected [32]. Even though infection with a single HPV type was common in this study the prevalence of multiple infection was high considering the fact that the cervical biopsy specimen was used, and this was more commonly observed among HR-HPV than in probable/LR-HPV types. HPV multiple infections is reported to be higher when cervical cells are used as the specimen than when the biopsies/tissue is used [33]. According to Guan et al. (2012) using cervical biopsies tends to reduce HPV multiple infection prevalence and increase the focus on HPV types causally associated with the observed lesion [33]. However, in this study, the high prevalence of multiple HPV types even when biopsies were used could be due to cross-contaminated during specimen collection because of the high prevalence of multiple HPV types in the South African population [34,35].

HR-HPV prevalence was high and increasing with increasing cervical disease [21,36,37]. Even though LR-HPV types were detected, they were more commonly detected as multiple HPV infections with other HR, probable and/or LR-HPV types. It is important to note that among the types not targeted by current commercial HPV vaccines but commonly detected in cervical cancer cases among women of African ancestry origin [22], HPV-35 was detected in 17.4% of the study population and appeared as a single infection in 7.9%. The prevalence of HPV-35 among women with CIN3 was similar to the one reported by Taku et al. (2021) in the same Province. Rad et al. (2017) reported HPV-35 as the fourth most common HPV type among South African women with cervical cancer [36]. Similarly, Denny et al. reported HPV-35 (9.7%) as the fourth most dominant type among women from South Africa, Ghana, and Nigeria with invasive cervical cancer [21]. In a meta-analysis study, Clifford et al. (2016) also report HPV-35 as the fourth most dominant type among African women with invasive cervical cancer regardless of HIV status [38]. Among Botswana women with CIN2 or CIN3, an HPV-35 prevalence of 40.0% has been reported [39]. The current HPV vaccines do not target HPV-35; the addition of HPV-35 to the Gardasil®9 types would increase the protection against HPV-associated diseases among women of African ancestry [22].

HPV-52 was found to be the sixth most dominant type in this population; however, it could be underestimated because, in cases of co-infection with HPV-33, -35, or -58, the Roche HPV genotyping assay used in the study cannot determine if HPV-52 is also present. It is therefore important to further investigate HPV-52 prevalence in this population. It is acknowledged that the study population does not represent the population of Eastern Cape Province and cannot be generalised. Despite these limitations, the information reported remains important for this province and South Africa as there is currently limited HPV information on this population.

## Conclusion

High overall HPV and HR-HPV prevalence were observed. HR-HPV prevalence was significantly increasing with increasing cervical intraepithelial lesion grades. HPV-35 was among the most commonly detected HPV types. The current HPV vaccines do not target HPV-35; the addition of HPV-35 to the Gardasil®9 types would increase the protection against HPV-associated diseases among women of African ancestry. This data will provide the National Department of Health with crucial HPV prevalence and distribution of HPV genotypes data among non-HPV vaccinated women in the Eastern Cape Province of South Africa.

## Supporting information

S1 File (XLSX)
